# FDG PET based prediction of response in head and neck cancer treatment: Assessment of new quantitative imaging features

**DOI:** 10.1371/journal.pone.0215465

**Published:** 2019-04-19

**Authors:** Reinhard R. Beichel, Ethan J. Ulrich, Brian J. Smith, Christian Bauer, Bartley Brown, Thomas Casavant, John J. Sunderland, Michael M. Graham, John M. Buatti

**Affiliations:** 1 Department of Electrical and Computer Engineering, The University of Iowa, Iowa City, United States of America; 2 Department of Internal Medicine, The University of Iowa, Iowa City, United States of America; 3 Department of Biostatistics, The University of Iowa, Iowa City, United States of America; 4 Department of Radiology, The University of Iowa, Iowa City, United States of America; 5 Department of Radiation Oncology, The University of Iowa, Iowa City, United States of America; North Shore Long Island Jewish Health System, UNITED STATES

## Abstract

**Introduction:**

18 F-fluorodeoxyglucose (FDG) positron emission tomography (PET) is now a standard diagnostic imaging test performed in patients with head and neck cancer for staging, re-staging, radiotherapy planning, and outcome assessment. Currently, quantitative analysis of FDG PET scans is limited to simple metrics like maximum standardized uptake value, metabolic tumor volume, or total lesion glycolysis, which have limited predictive value. The goal of this work was to assess the predictive potential of new (i.e., nonstandard) quantitative imaging features on head and neck cancer outcome.

**Methods:**

This retrospective study analyzed fifty-eight pre- and post-treatment FDG PET scans of patients with head and neck squamous cell cancer to calculate five standard and seventeen new features at baseline and post-treatment. Cox survival regression was used to assess the predictive potential of each quantitative imaging feature on disease-free survival.

**Results:**

Analysis showed that the post-treatment change of the average tracer uptake in the rim background region immediately adjacent to the tumor normalized by uptake in the liver represents a novel PET feature that is associated with disease-free survival (HR 1.95; 95% CI 1.27, 2.99) and has good discriminative performance (c index 0.791).

**Conclusion:**

The reported findings define a promising new direction for quantitative imaging biomarker research in head and neck squamous cell cancer and highlight the potential role of new radiomics features in oncology decision making as part of precision medicine.

## Introduction

Quantitative imaging with 18 F-fluorodeoxyglucose (FDG) PET is routinely performed for head and neck squamous cell cancer (HNSCC) patients, yet the information used from these scans is often limited to qualitative analysis determining the presence or absence of disease in an anatomically defined area along with a report of the maximum standardized uptake value (SUV_max_). While visual analysis is sufficient for diagnosis and staging, a more quantitative approach to FDG PET/CT analysis holds promise as a predictive tool. For example, a recent review paper by Castelli et al. [1] summarized the results of 45 studies (overall 2928 patients) regarding the predictive value of FDG PET with respect to clinical outcome in head and neck cancer treatment with chemoradiotherapy (CRT). The vast majority of the investigated studies were focused on simple, standard quantitative indices like SUV_max_, peak uptake value (SUV_peak_) [2], metabolic tumor volume (MTV), and Total Lesion Glycolysis (TLG); only three studies performed texture or shape analysis. The study concluded that MTV and TLG in pre-treatment PET scans showed good correlation with disease free survival (DFS) or overall survival (OS), while simple indices like SUV_max_ and SUV_peak_ showed less promise [1].

Quantitative imaging biomarkers (QIBs) represent an underutilized component of precision medicine. Radiomic feature analysis may define useful characteristics of tumors before, during, and after treatment, not revealed by conventional (quantitative) assessment. The importance of QIBs is also underlined by the Radiological Society of North America’s Quantitative Imaging Biomarkers Alliance (QIBA) [3] initiative, which seeks to improve and define validation methods whereby the value and practicality of quantitative imaging biomarkers can be realized by reducing variability across devices, patients, analysis methods, and time.

In this paper, we investigate the association of 17 new (i.e., nonstandard) quantitative imaging features in HNSCC with DFS and compare them against 5 standard features (i.e., Max, Peak, Mean, MTV, and TLG), resulting in a total of 22 features analyzed. Our work is motivated by the emergence of efficient tools for semi-automated segmentation of lesions in FDG PET scans (e.g., Beichel et al. [4]), which help facilitate the process of quantitative image feature calculation in a clinical setting. The goal of this work is to identify promising features that can be calculated from semi-automated lesion segmentations so that they can be further evaluated in subsequent large (multi-site) studies, and eventually, will result in novel biomarkers that are approved for clinical use.

## Methods

### Data

For this study, which was approved by the Institutional Review Board, FDG PET/CT scans from 58 subjects diagnosed with HNSCC from 2004–2008 were available for retrospective data analysis. This population was selected because of the availability of both imaging and long-term clinical follow-up data, which enables assessing the prognostic characteristics of features. Descriptive statistics of the data are presented in Table 1. We note that while standard clinical features like age, gender, smoking status were available for the studied subjects, they did not appear to add predictive power to the radiomics features presented. Furthermore, image data as well as corresponding clinical metadata (sex, age, smoking status, drinking history, stage, primary site location, etc.) are part of a head and neck cancer data collection available on NCI’s The Cancer Imaging Archive in DICOM format [5, 6]. In addition, it is noteworthy that the patients were largely accrued prior to the recognition of HPV as an important prognostic variable and hence this laboratory value was not available for the vast majority of the cohort. Furthermore, the majority of cancers were treated with primary chemo-radiotherapy. Chemotherapy was platinum based and given either weekly or on an every three week concomitant schedule. Primary cancers included the following anatomical regions: base of tongue, oropharynx, pyriform sinus, tonsil, hypopharynx, and nasopharynx. The cases had varying T and N stage, but no distant metastases. Two FDG PET/CT scans were analyzed for each subject—a pre-treatment and a post-treatment scan. Patients were treated after full evaluation at an interdisciplinary tumor board, with most receiving definitive chemoradiotherapy. A PET/CT for response assessment was obtained at 8–12 weeks after completion of radiation therapy [7, 8]. For imaging, the clinical standard protocol was used and performed on Siemens Biograph 40, Siemens Biograph Duo, or GE Medical Systems Discovery LS PET/CT scanners. All subjects were injected with 370 MBq ± 10% of [F-18]FDG with an uptake time of 90 minutes ± 10%. In all cases subjects were fasted for >4 hours and had blood glucose <200 mg/dL. Because of the interest in the H&N region, patients were imaged with arms down, and CT-based attenuation correction was performed. All reconstructions were performed with iterative 2D OSEM algorithms. The voxel size ranged from 3.4×3.4×2.0 to 4.3×4.3×5.0 mm, with the majority at 3.5×3.5×3.4 mm.

**Table 1:** Demographic and clinical characteristics.

Patient Characteristics		N (%)
Median Age at Diagnosis (range)		55 (21–80)
Sex		
	Males	47 (81.0)
	Females	11 (19.0)
Primary Site		
	Tonsil	24 (41.4)
	Base of Tongue	22 (37.9)
	Oropharynx	5 (8.6)
	Nasopharynx	3 (5.2)
	Hypopharynx	2 (3.4)
	Pyriform Sinus	2 (3.4)
T Stage		
	2	29 (50.0)
	3	11 (19.0)
	4	2 (3.4)
	4a	12 (20.7)
	4b	4 (6.9)
N Stage		
	0	5 (8.6)
	1	9 (15.5)
	2a	3 (5.2)
	2b	20 (34.5)
	2c	17 (29.3)
	3	4 (6.9)
Median Follow-up Months (range)		48.8 (5.4–124.3)
Recurrence or Death		25 (43.1)

### Calculation of quantitative imaging features

**Fig 1:**
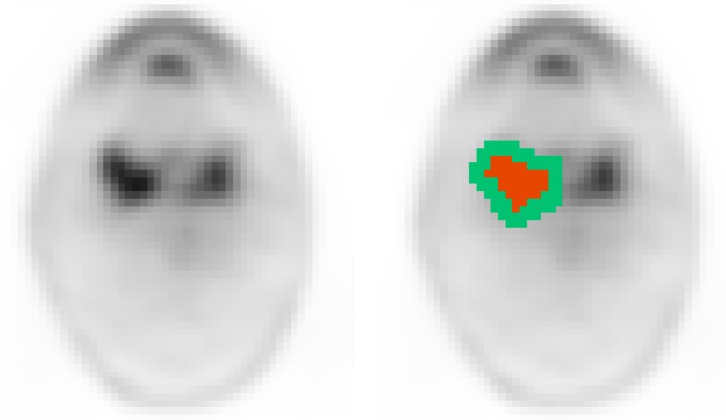
Example of a rim region (green mask) used for calculating feature RA relative to the lesion segmentation (red mask).

First, all FDG PET volumes were SUV normalized by utilizing the PET DICOM Extension [9] for 3D Slicer, a multi-platform free and open software package for visualization and medical image computing [10]. Second, an experienced radiation oncologist inspected all scans and identified primary tumors, which were segmented by utilizing the semi-automated segmentation approach described by Beichel et al [4]. The approach utilizes a graph-based optimization algorithm for segmentation, requires little user interaction, and is available for 3D Slicer in the form of an editor effect extension [11]. All segmentations were performed following standard clinical practice in radiation oncology. Note that out of 58 cases, 25 showed residual uptake in the primary lesion on the 8–12 week follow-up PET scan. These lesions were also segmented using the same segmentation tool. Consequently, no segmentation was performed for 33 follow-up PET scans due to complete response after treatment. Third, the five most commonly utilized basic and 17 new features were calculated utilizing the PET-IndiC extension for 3D Slicer [12]. An overview and detailed description of all calculated features is given in Table 2. The majority of new features were designed to characterize the SUV uptake pattern within the segmented lesion by utilizing descriptive statistics. One exception is the feature SAM (standardized added metabolic activity), which was proposed by Mertens et al. [13] with the goal to develop a partial volume independent marker of total lesion glycolysis. SAM utilizes a small rim region around the segmented lesion for partial volume correction. Note that Mertens et al. [13] proposes two versions: SAM and normSAM. In this work we utilize SAM, and similarly as most of the other features listed in Table 2, we have normalized this feature by the uptake in the liver as described below in more detail. Furthermore, we have included the average SUV of this rim region as a separate feature, which was dubbed RA. An example for the rim around a lesion is given in Fig 1. Fourth, to account for different metabolic base-line activity, we further normalized SUV measurements to the metabolic activity of the liver at the time of imaging, similarly as proposed by Wahl et al.[2]. Such an approach is especially important for change assessment using pre- and post-treatment PET scans. For normalization to metabolic baseline activity, a spherical liver measurement region was automatically defined by utilizing the approach described by Bauer et al.[14], which is also available in 3D Slicer in the form of an extension [15]. From this liver measurement region, the average SUV is calculated, and all SUV-based features were normalized by dividing them by this uptake value. For cases with pre-treatment (t_0_) and post-treatment (t_1_) segmentations, the change of the quantitative image features was calculated as $\Delta QIF={QIF}_{t_{0}}-{QIF}_{t_{1}}$.

**Table 2:** Overview of image-derived features utilized.

Feature	Type	Description	Liver uptake normalization
Max	S	Maximum value in region of interest (-)	Y
Peak	S	Maximum average gray value that is calculated from a 1 cubic centimeter sphere placed within the region of interest [2] (-)	Y
Mean	S	Mean value in region of interest (-)	Y
MTV	S	Volume of region of interest (ml)	N
TLG	S	Total lesion glycolysis (ml)	Y
Min	A	Minimum value in region of interest (-)	Y
Std	A	Standard deviation in region of interest (-)	Y
RMS	A	Root-mean-square value in region of interest (-)	Y
First Quartile	A	25th percentile value in region of interest (-)	Y
Median	A	50th percentile value in region of interest (-)	Y
Third Quartile	A	75th percentile value in region of interest (-)	Y
Upper Adjacent	A	First value in region of interest not greater than 1.5 times the interquartile range (-)	Y
Q1 Distribution	A	Percent of gray values that fall within the first quarter of the grayscale range within the region of interest (%)	N
Q2 Distribution	A	Percent of gray values that fall within the second quarter of the grayscale range within the region of interest (%)	N
Q3 Distribution	A	Percent of gray values that fall within the third quarter of the grayscale range within the region of interest (%)	N
Q4 Distribution	A	Percent of gray values that fall within the fourth quarter of the grayscale range within the region of interest (%)	N
Glycolysis Q1	A	Lesion glycolysis calculated from the first quarter of the grayscale range within the region of interest (ml)	Y
Glycolysis Q2	A	Lesion glycolysis calculated from the second quarter of the grayscale range within the region of interest (ml)	Y
Glycolysis Q3	A	Lesion glycolysis calculated from the third quarter of the grayscale range within the region of interest (ml)	Y
Glycolysis Q4	A	Lesion glycolysis calculated from the fourth quarter of the grayscale range within the region of interest (ml)	Y
SAM	A	Standardized added metabolic activity [13] (ml)	Y
RA	A	Rim average; mean of uptake in a 2 voxel wide rim region around region of interest (-)	Y

Feature type: S… standard and A… new.

### Statistical analysis

Survival data analysis methods were used to assess the effect of each feature on DFS. DFS was defined as time from initial treatment to disease recurrence or death. Patients for whom events were not observed during their follow-up were treated as censored observations at the date of last follow-up in the analysis. Descriptive survival probabilities were estimated and plotted with the method of Kaplan-Meier for patients grouped into high, intermediate, and low imaging feature categories according to tertiles. Quantitative imaging feature effects on survival were modeled individually with Cox regression. Regression estimates are reported as hazard ratios (HRs) for one standard deviation increases in the imaging feature along with 95% confidence intervals (CIs). Rank-order agreement (concordance) between prognostic scores from the regression models and observed survival times was estimated with the c index [16]. This index can be interpreted as the probability that, of two randomly selected patients, the one with a better prognostic score will survive longer. Values of 0 or 1 indicate perfect concordance and 0.5 no concordance. Two-sided p-values for tests of the significance of features in the regression models are reported, unadjusted for the number of tests performed. To account for multiple statistical testing, false-discovery rate (FDR) was also computed with the method of Benjamini and Hochberg [17]. FDR can be used to select among the full set of features tested so as to control the expected proportion of false positive statistical findings. Features were identified as significant in this study at a FDR of 10%. Statistical analysis was performed with the survival [18] and survminer [19] packages in version 3.4.1 of the R statistical software [20].

## Results

Patients were followed for a median of 48.8 months (range 5.4–124.3 months). DFS events (recurrence or death) were observed for 25 of the 58 patients with baseline features and 13 of the 25 with post-treatment change features. Hence, 13 of 25 positive scans at 8–12 week follow-up had a true recurrence or died and 12 of 25 had false positive initial follow-up scans and did not have evidence of recurrence with follow-up.

**Fig 2:**
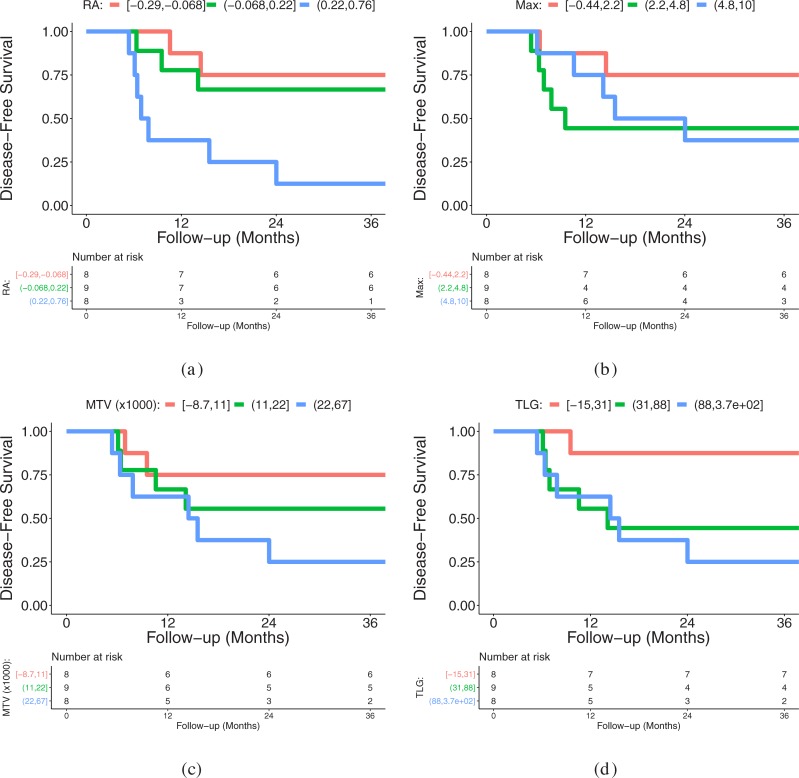
Kaplan-Meier plot of disease-free survival for patients stratified into low, intermediate, and high groups of post-treatment change in RA in image feature using tertiles. (a) Plot for RA. (b) Plot for Max. (c) Plot for MTV. (d) Plot for TLG.

Tables 3 and 4 provide a summary of hazard ratios with corresponding CIs, univariate Cox regression p-values, false-discovery rates, as well as c index values for features at baseline and post-treatment change, respectively. Based on the results, imaging features with unadjusted p-values less than 5% include baseline MTV, Glycolysis Q2, and RA (rim average); and post-treatment change in RA, Min, and 1st Quartile features. After p-value adjustments for the multiple statistical tests performed, post-treatment change in RA stands out as being significant at a 10% FDR. Its estimated hazard ratio indicates a 95% increase in the rate of recurrence or death for a one standard deviation increase in this imaging feature. The c index value of 0.791 signifies moderately high concordance between the feature’s estimated survival effect and observed survival. Compared to the available clinical features, only T Stage was found to be significantly associated with DFS, but with a smaller c index of 0.69 in the post-treatment change cohort. Fig 2 provides a comparison of Kaplan-Meier curves for features RA, Max, MTV, and TLG in which RA stands out as having a consistent increasing trend in survival across its categories.

**Table 3:** Univariate analysis at baseline. Features are sorted by p-values.

Feature	HR (95% CI)	p-value	FDR	c Index
MTV	1.49 (1.05, 2.12)	0.027	0.316	0.647
Glycolysis Q2	1.41 (1.03, 1.94)	0.034	0.316	0.645
RA	1.41 (1.01, 1.97)	0.043	0.316	0.634
TLG	1.37 (0.98, 1.92)	0.065	0.321	0.639
Glycolysis Q1	1.37 (0.97, 1.93)	0.077	0.321	0.637
Glycolysis Q3	1.33 (0.96, 1.86)	0.087	0.321	0.638
1st Quartile	1.33 (0.92, 1.92)	0.133	0.346	0.609
SAM	1.30 (0.92, 1.82)	0.134	0.346	0.637
Min	1.29 (0.91, 1.84)	0.151	0.346	0.631
Q4 Distribution	1.32 (0.89, 1.95)	0.165	0.346	0.543
Glycolysis Q4	1.27 (0.90, 1.78)	0.173	0.346	0.634
Median	1.28 (0.87, 1.88)	0.211	0.368	0.588
Q1 Distribution	0.78 (0.53, 1.16)	0.217	0.368	0.564
Q3 Distribution	1.25 (0.84, 1.85)	0.273	0.429	0.535
Mean	1.22 (0.83, 1.78)	0.311	0.455	0.573
3rd Quartile	1.20 (0.82, 1.76)	0.356	0.489	0.566
RMS	1.18 (0.81, 1.72)	0.385	0.498	0.560
Peak	1.14 (0.79, 1.66)	0.481	0.588	0.566
Upper Adjacent	1.11 (0.76, 1.62)	0.591	0.662	0.558
Max	1.10 (0.76, 1.60)	0.602	0.662	0.565
Std	1.06 (0.72, 1.55)	0.769	0.793	0.547
Q2 Distribution	1.05 (0.71, 1.55)	0.793	0.793	0.503

**Table 4:** Univariate analysis of post-treatment change in features. Features are sorted by p-values.

Feature	HR (95% CI)	p-value	FDR	c Index
RA	1.95 (1.27, 2.99)	0.002	0.048	0.791
Min	1.81 (1.07, 3.05)	0.027	0.206	0.730
1st Quartile	1.58 (1.02, 2.46)	0.041	0.206	0.707
Q4 Distribution	2.10 (1.00, 4.42)	0.050	0.206	0.667
Median	1.55 (0.98, 2.46)	0.059	0.206	0.689
Mean	1.54 (0.97, 2.46)	0.068	0.206	0.667
RMS	1.53 (0.96, 2.45)	0.076	0.206	0.658
3rd Quartile	1.53 (0.95, 2.47)	0.079	0.206	0.644
Peak	1.50 (0.93, 2.42)	0.093	0.206	0.644
MTV	1.45 (0.90, 2.31)	0.124	0.206	0.653
Max	1.46 (0.90, 2.37)	0.125	0.206	0.622
Glycolysis Q4	1.43 (0.90, 2.28)	0.130	0.206	0.671
Glycolysis Q1	1.39 (0.90, 2.16)	0.138	0.206	0.685
Upper Adjacent	1.41 (0.88, 2.26)	0.149	0.206	0.622
Q1 Distribution	0.63 (0.34, 1.18)	0.150	0.206	0.626
TLG	1.37 (0.89, 2.10)	0.153	0.206	0.671
Std	1.44 (0.87, 2.38)	0.159	0.206	0.617
Glycolysis Q2	1.32 (0.88, 1.96)	0.174	0.213	0.653
SAM	1.31 (0.85, 2.01)	0.215	0.239	0.671
Glycolysis Q3	1.30 (0.86, 1.98)	0.217	0.239	0.662
Q3 Distribution	1.20 (0.66, 2.19)	0.550	0.577	0.509
Q2 Distribution	1.09 (0.63, 1.88)	0.747	0.747	0.590

## Discussion

Development of image-based biomarkers is a multi-step process that begins at discovery and migrates to validation and ultimately to regulatory approval with many steps in between [21]. While offering great potential for clinical use, the development of biomarkers is resource intensive and requires development of practical tools for consistent definitions of regions of interest, normalization, and feature calculation, similar to the requirement for development of rapid sequencing technologies for molecularly-based precision techniques. Thus, the goal of this work was to identify new FDG PET based features that are promising for outcome prediction in HNSCC, and therefore, should be further investigated by the community.

### Survival associations and impact

Results of pre-treatment FDG PET scans confirm the findings reported by Castelli et al.[1]; MTV and TLG perform better than max and peak markers. In this context, note that the number of papers utilizing the peak marker included in the review performed by Castelli et al. [1] is limited. In our study, we opted to normalize tracer uptake by the average liver uptake. Omitting this step would lead to a similar ranking (MTV: p = 0.0283, TLG: p = 0.1464, Peak: p = 0.8292, and Max: p = 0.9764). A potential reason for the better relative performance of MTV and TLG could be that MTV and TLG are spatially more “inclusive” than max and peak measurements, accounting for the full volumetric and metabolic extent of the tumor, rather than a simple assessment of the highest level of metabolic activity. The new features RA and Glycolysis Q2 showed similar performance to MTV and TLG on pre-treatment scans. Because multiple statistical testing is employed, we report p-values adjusted to control the false-discovery rate (FDR), and a FDR of 10% was specified in this study for the determination of statistical significance. Based on this criterion, none of the features at baseline is deemed to be significant due to the number of features investigated combined with the limited dataset size.

When looking at the survival effect of post-treatment feature value change in those selected cases where primary lesions were still visible in the follow-up FDG PET scan, it is important to seek potential imaging based biomarkers that might distinguish the high proportion of false positive scans from those indicating true recurrence. In our analysis, RA is ranked highest (FDR: 0.048) and found to be significant at a 10% FDR. This is a particularly challenging group since those with residual uptake have approximately a 50% chance of having residual disease (true positive) versus 50% with no true recurrence that are false positives. In this setting, MTV and TLG were not found to statistically distinguish between true and false positives.

**Fig 3:**
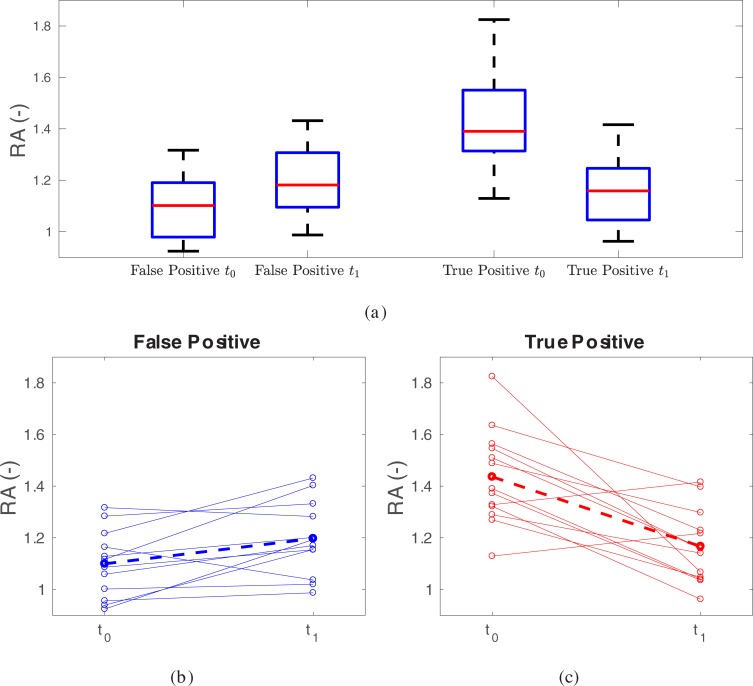
Summary of RA values at time points *t*_0_ (pre-treatment) and *t*_1_ (post-treatment). (a) Boxplots. (b) False positive cases and (c) true positive cases. The dashed line indicates the average trend.

We speculate that this finding can be explained as follows. The region of interest for RA is defined as the two voxels wide rim surrounding the segmented tumor volume. Hence, RA captures the metabolic activity in the primary tumor’s periphery (Fig 1). Typically, cases with recurrence had higher RA-values in the pre-treatment scan compared to the post-treatment scans (positive $\Delta RA={RA}_{t_{0}}-{RA}_{t_{1}}$), indicating a loss of activity in this rim region potentially due to low or reduced immunogenicity in the tumor. Patients with false positive scans tended to have a lower RA to start and tended to see an increase in RA with treatment, which appeared to portend a better outcome (negative $\Delta RA={RA}_{t_{0}}-{RA}_{t_{1}}$) perhaps due to enhanced inflammation or immunogenicity in this region. The suggestion of less inflammation with recurrence highlights the complex interplay between radiation, chemotherapy and tumor. A summary of RA pre- and post- treatment trend patterns is provided in Fig 3. This suggests that an increase in activity in this peripheral region is predictive for improved outcome and we hypothesize this is consistent with recently supported data showing that increased immune response to tumors portends better outcomes [22–25]. Furthermore, it may help differentiate between true positive recurrences from false positive results that have been a common issue in post-treatment PET-imaging [7, 26–31]. Similarly, closely nearby-ranked features Minimum and 1st Quartile uptake, also capture aspects of the tracer uptake in the region of transition from hot lesion core to background, especially in the case of non-necrotic lesions, which represents the vast majority of cases investigated in this work (Fig 1). This is also reflected in the correlation of features Minimum and 1^st^ Quartile with RA (0.8022 and 0.8496, respectively). In this context, there are several issues to consider. First, out of these three features, only RA has a FDR below 10%. Second, the feature Minimum is quite sensitive to image noise when compared to RA, and thus, using Minimum is not recommended. Third, while there is room for optimization of features like RA and 1st Quartile their results point in the same direction. Furthermore, standard features (e.g., max, peak, MTV, or TLG) were not significantly associated with survival when looking at post-treatment feature change.

When considering both pre-treatment and change at post-treatment, RA is the only feature that has a p-value<0.05 before FDR correction. In addition, when correcting for FDR, it stays significant as a post-treatment change based feature. Therefore, we conclude that RA represents a good candidate for further investigations. Clearly, RA is functionally the reverse of what is currently broadly utilized by the community (e.g., SUV_max_, MTV, TLG) as it does not focus on the core region of the lesion, but instead on the periphery. In this context, one exception is the feature SAM, which corrects for partial volume effects by utilizing the tumor rim region [13].

Based on our findings, we conducted a literature search to see whether similar features were investigated for the purpose of outcome prediction. We found one recent publication that describes a tumor shell-based radiomics approach, which analyzes the tumor periphery [32]. The shell feature captures a sequence of morphologic patterns across the primary tumor boundary, such as shape, size, SUV values, and heterogeneities in a simple 2D map, which is vectorized to form a feature vector and is subsequently used as the input for a support vector machine (SVM) classifier. In [32], the authors demonstrated the ability of this approach in predicting treatment response based on pre-treatment PET scans for patients receiving stereotactic body radiation therapy (SBRT) for early stage non-small cell lung cancer and for patients receiving external beam radiation therapy (EBRT) and concurrent chemotherapy followed by high-dose-rate intracavitary brachytherapy (ICBT) in stage IB-IVA cervix cancer. While the work reported in [32] uses a different approach for deriving tumor periphery features and assesses them in two different applications, we conclude that the lesion rim is promising for future investigations. Furthermore, the increased activity in the periphery of lesions could represent enhanced immune response, which could have potential utility in determining activity of immune therapy interventions. So far, assessing therapy response from these agents has been difficult and hence the impact could be substantial, but requires significant investigation. In addition, future histologic evaluation of these rim regions is needed to confirm or refute this potential explanation.

### Future work

The goal of this work was to identify potentially promising quantitative imaging features. As such, the presented work is part of an ongoing evaluation process, following the consensus approach outlined in [21]. In our future work, we will assess the impact of imaging equipment, reconstructions, as well as the acquisition settings on feature performance. Also, some features might be more sensitive to variability in lesion segmentation than others, and the impact of rim thickness on predictive performance will be investigated. In this work, only the primary lesion was considered. However, efficient PET image segmentation tools like the one presented in Beichel et al. [4] make it feasible to individually segment involved lymph nodes. At this stage, it is unclear how to best utilize this additional information and if this could potentially add predictive value. Furthermore, in future work we plan on increasing our evaluation database considerably and studying the impact of different uptake reference regions (aortic arch, cerebellum, etc.) for normalization on prediction performance of features.

## Conclusion

The promise of precision medicine is more than the potential to use genetic information, but also includes identification of image-based biomarkers that define useful characteristics of tumors before, during, and after treatment. The presented work compares standard and new features and assesses their suitability for FDG PET based prediction of response in head and neck cancer treatment. The reported findings should help define potential new directions for biomarker research in HNSCC.

## Supporting information

S1 File Table with quantitative PET imaging features and outcome data listed per subject studied. (CSV)
